# Mothers, fathers, sons, and daughters: gender differences in factors associated with parent-child communication about sexual topics

**DOI:** 10.1186/1742-4755-7-31

**Published:** 2010-12-14

**Authors:** Ellen K Wilson, Helen P Koo

**Affiliations:** 1 RTI International, 3040 Cornwallis Road, PO Box 12194, Research Triangle Park, NC 27709, USA

## Abstract

**Background:**

In the United States, nearly half of high school students are sexually active, and adolescents experience high rates of unintended pregnancy and sexually transmitted diseases. Parents can have an important influence on their children's sexual behaviour, but many parents do not talk with their children about sexual topics. Research has shown significant differences in parent-child communication about sexual topics depending on the gender of both the parent and the child. Little is known, however, about the reasons for these gender differences. The purpose of this paper is to describe how factors associated with parent-child communication about sexual topics differ by gender.

**Methods:**

Data are from a nationwide online survey with 829 fathers and 1,113 mothers of children aged 10 to 14. For each of the four gender groups (fathers of sons, fathers of daughters, mothers of sons, mothers of daughters), we calculated the distribution of responses to questions assessing (1) parent-child communication about sex-related topics, and (2) factors associated with that communication. We used chi-square tests to determine whether the distributions differed and the false discovery rate control to reduce the likelihood of type I errors.

**Results:**

With both sons and daughters, fathers communicated less about sexual topics than mothers did. Fathers also had lower levels of many characteristics that facilitate communication about sex (e.g., lower self-efficacy and lower expectations that talking to their children about sex would have positive outcomes). Compared with parents of sons, parents of daughters (both mothers and fathers) talked more about sexual topics, were more concerned about potential harmful consequences of sexual activity, and were more disapproving of their child having sex at an early age.

**Conclusions:**

Using a large national sample, this study confirms findings from previous studies showing gender differences in parent-child communication about sexual topics and identifies gender differences in factors that may influence parent-child communication about sexual topics. Interventions designed to support parent-child communication about sexual topics should emphasize to both mothers and fathers the importance of talking to sons as well as daughters. Fathers need particular support to overcome the barriers to communication they encounter.

## Background

Despite recent declines in rates of sexual activity among teens and preteens in the United States, in 2009 nearly one-half (46%) of high school students reported having had sexual intercourse, and 6% reported having had sexual intercourse for the first time before age 13 years [1]. Because many adolescents are unprepared to protect themselves from the potential negative consequences of sexual activity, they experience high rates of unintended pregnancy and sexually transmitted diseases (STDs). In the United States, the rate of unintended pregnancy for sexually active girls aged 15 to 17 is 146 per 1000, compared with 69 per 1000 for sexually active women of all ages (15 to 44) [2]. Teens are also disproportionately infected with STDs: although sexually active teens aged 15 to 19 and young adults aged 20 to 24 represent just 25% of the sexually experienced population, they acquire nearly half of all new STDs each year [3].

Research has shown that parents can have an important influence on their children's sexual behavior [4,5], and most adolescents and adults agree that it would be easier for adolescents to postpone sexual activity and avoid pregnancy if they were able to have more open, honest conversations with their parents about these topics [6]. Many parents, however, either do not talk to their children about sex at all or have only limited communication on the topic [4]. How much parents talk about sex and what topics they address have been found to differ substantially by the gender of both the parents and the children. Generally, parents are more likely to talk about sexual topics with the same-sex child: fathers are more likely to talk with their sons than their daughters, and mothers are more likely to talk with their daughters than their sons [4,7-9]. However, mothers generally talk more than fathers to both sons and daughters about most sexual topics [4,7,9,10]. These findings suggest that adolescents of both genders may receive inadequate guidance about sexual topics from their fathers, and sons in particular may not receive adequate guidance from either parent.

Little is known, however, about the reasons for these gender differences in communication. Research on family relations has documented differences in parent-child relationships according to the gender of both parent and child [11-13]. For example, mothers tend to be more involved with teaching their children than fathers, children tend to feel closer to their mothers than their fathers, and parents are more likely to spend time with the same-sex child [11]. This research does not investigate gender-based differences specifically related to parent-child communication about sexual topics. A few qualitative studies provide some insight, suggesting that parents may feel more comfortable talking to a same-sex child than an opposite-sex child because they feel more knowledgeable about what a same-sex child is experiencing [9,14]; that, for fathers, the potential negative consequences of sex are a greater concern for daughters than for sons [15]; and that fathers feel they are less effective communicators than mothers--in part because they spend less time with their children and are less in tune with them [9,16]. Although numerous quantitative studies have identified factors associated with parent-child communication about sex [4,17-20], none have explored how these factors may differ by either the gender of the parent or the gender of the child.

To address this gap, this study uses a large national dataset to describe how factors likely to influence parent-child communication about sexual topics differ by gender. To identify relevant factors, it draws on findings from previous research and the framework of the health belief model [21], which suggest that parents' communication with their children about sexual topics would be influenced by (1) their perception of the threat to their children from sex-related risks, (2) their perception of the benefits of talking to their children about sex, and (3) the barriers and facilitators to such communication. Identification of differences in these factors across the four gender groups (fathers of sons, fathers of daughters, mothers of sons, and mothers of daughters) can improve understanding of the reasons for gender differences in parent-child communication about sexual topics and thus inform the design of interventions to overcome barriers to communication specific to each of the four gender groups.

## Methods

### Sampling Recruitment and Data Collection

This study was conducted as part of an evaluation of the Parents Speak Up National Campaign (PSUNC), a media campaign funded by the U.S. Department of Health and Human Services. The objective of the campaign is to encourage parents to talk to their children early and often about delaying sexual activity. The campaign includes print, radio and television advertisements; a Web site; and activities organized by outreach centers targeting different racial and ethnic groups.

Data for this analysis come from the baseline survey of the PSUNC Parent Efficacy Study, a randomized controlled experiment conducted with parents of children between 10 and 14 years of age [22]. The data were collected between August and October 2007 through an online questionnaire. All study procedures and materials were reviewed and approved by the Institutional Review Board of RTI International.

Participants were recruited from the Knowledge Networks online research panel, the only online research panel in the United States that is based on probability sampling and includes both online and offline populations. The panel is recruited using random-digit-dialing sampling from a sample frame consisting of the entire U.S. residential telephone population. Telephone numbers in telephone exchanges with a higher proportion of black or Hispanic households have a higher probability of being selected than households in exchanges with a lower proportion of black or Hispanic households. Individuals who do not have a computer and access to the Internet are provided MSN TV service and free Internet access, which allows representation of both online and offline households. More detailed information about the Knowledge Networks sample is available at the Knowledge Networks Web site http://www.knowledgenetworks.com/ganp/docs/knowledge%20networks%20methodology.pdf.

For this study, we identified all adult panelists living with at least one child between 10 and 14 years of age (*N *= 3,217). We focused on the parents of children in this age range because they were the target audience for the campaign. Mothers and fathers were sampled separately and were not from the same family. If parents had more than one child aged 10 to 14, one of those children was randomly selected, and all subsequent questions focused on that child. A total of 2,439 parents (75.8%) responded to the study invitation and were confirmed as eligible. Of these, 1,969 parents (80.7%: 1,125 mothers and 844 fathers) completed the baseline survey. Study recruitment and eligibility are described elsewhere [22].

For the efficacy study, parents were randomly assigned to treatment and control conditions, where treatment consisted of exposure to PSUNC advertisements and print materials, and control consisted of no exposure to PSUNC messages. At the time of the baseline survey, however, neither the treatment nor control groups had been exposed to PSUNC messages.

### Measures

The survey collected data on sociodemographic characteristics, parents' communication with their child about a range of topics related to sexuality, and factors hypothesized to influence parent-child communication about sexual topics. We used eight measures of communication about sex. One measure assessed whether the parent had asked or recommended that their child wait to have sex, with response options of "yes" or "no." The other seven measures assessed how much parents had talked with their child about each of seven specific topics (e.g., biology of sex and pregnancy, issues about dating and relationships, whether to wait to be sexually active until you are married); response categories ranged from 1 (*a great deal*) to 4 (*not at all*) [23].

Drawing on the health belief model, the factors we hypothesized would influence communication were parents' perception of the threat of early sexual intercourse (their attitudes toward teen sex and their perception of the likelihood their child will have sex as a young teen); the outcomes they expect from talking about sex with their child; and barriers and facilitators to communication, including characteristics of their relationship with their child, self-efficacy, specific barriers to communication, and their beliefs about the right developmental time for talking about sex. The measures for these factors are described in detail in Table 1.

**Table 1:** Measures of Potential Influences on Communication

Measure and number of items	Question	Sample items	Response categories
**Measures of perception of threat**			

Parent attitudes toward teen sex (2 items)	For each of the following, please indicate how much you agree or disagree with the statement.	Sexual activity is likely to have harmful psychological and physical effects for teens. You disapprove of {child name} being sexually active as a young teenager.	*1 (strongly agree) to 4 (strongly disagree)*

Perception of likelihood child will become sexually active as a young teen (1 item)	How likely do you think {child name} would be to be sexually active if asked by someone [he/she] was dating as a young teen?	-	*1 (very likely) to 4 (not at all likely)*

**Measures of perceived benefits of talking**			

Expectations of outcomes of talking about sex (6 items)	What are your expectations about talking with {child name}? If you talk early and often with {child name} about sexual topics (such as waiting to be sexually active until he/she is older). . .	. . . {child name} will be less likely to be sexually active as a young teen . . . {child name} will think you are a hypocrite	*1 (strongly agree) to 4 (strongly disagree)*

**Measures of barriers and facilitators to communication**			

Shared activities with child (6 items)	For the following list of activities, indicate whether this is something you and {child name} do together at least once a week, at least once a month, less often, or never.	Gone shopping Done homework or school projects when school is in session	*1 (at least once a week) to 4 (never)*

Closeness to child (5 items)	How often would it be true for you to make each of the following statements about {child name}?	You get along well with [him/her] {Child name} and you make decisions about [his/her] life together	*1 (always) to 4 (never)*

Conflict with child (1 item)	During the past 12 months, how often had you argued or had a fight with {child name}?	-	*1 (0 times) to 5 (10 or more times)*

Self-efficacy for communication about sex (4 items)	How sure are you that you can always explain to {child name} . . .	. . . why he/she should wait until he/she is older to be sexually active . . . how to make a [boy/girl] wait until [he/she] is ready to be sexually active	*1 (completely sure) to 7 (not sure at all)*

Barriers to communication about sex (3 items)	How much do you agree or disagree with each of the following statements?	You really don't know enough about sexual activity or waiting to have sex to talk about them with {child name}. It is easy for you to find the time to talk with {child name} about sexual activity and waiting to have sex.	*1 (strongly agree) to 4 (strongly disagree)*

Beliefs about timing of talking about sex (1 item)	How much do you agree or disagree with each of the following statements?	You are sure this is the right developmental time to talk with {child name} about sexual activity and waiting to have sex.	*1 (strongly agree) to 4 (strongly disagree)*

### Data Analysis

Because parents are likely to think and talk differently to their children about sex if they believe their child has already engaged in sexual activity, we excluded from the analysis the few parents (15 fathers; 12 mothers) who reported that they thought their child had already been sexually active. This left a total of 829 fathers and 1,113 mothers in the sample.

We used SAS for all analyses. For each of the four gender groups (fathers of sons, fathers of daughters, mothers of sons, mothers of daughters), we calculated the distribution of responses to each item and used chi-square tests to determine whether the distributions differed. For ease of presentation, we do not present the full distribution for each variable; rather, in both the table and the text, we present only the percentage who chose a response category at one end of the scale (e.g., the percentage who "strongly agree" with an item). Because we conducted multiple comparisons across the four groups, we used the false discovery rate control to reduce the likelihood of type I errors [24]. As a measure of the statistical significance of any differences between the four groups, we present the *q*-values (the false discovery rate analogue of *p*-values).

We also conducted multivariate analysis to determine whether the effects of any of the factors hypothesized to influence parent-child communication differed according to gender of the parent or the child. Because we did not find any significant gender differences in the effects of those factors, we do not present findings from the multivariate analysis here.

## Results

### Sample Description

The mean age of fathers in the study was 44.7 years, and the mean age of mothers was 42.1 years (Table 2). The mean age of the reference child was 12.2-12.3 years. Respondents' level of education was higher than the national average: Nearly one half had a college degree, a substantially higher proportion than the national figure of 25% of adults aged 25 or older [25]. Respondents were predominantly white (88.5% of fathers; 85.5% of mothers). Close to 90% of fathers in the study were married and living with their child's mother. Mothers were less likely to be in intact nuclear families: 75% were married, and 65% were living with their child's father.

**Table 2:** Sample Characteristics

	Fathers of sons (n = 455)	Fathers of daughters (n = 374)	Mothers of sons (n = 561)	Mothers of daughters (n = 552)
Parent age (mean years)	44.7	44.7	42.1	42.1
Child age (mean years)	12.3	12.2	12.3	12.2
Parent education (%)				
Less than high school	3.3	2.1	1.6	1.3
High school graduate	15.2	9.9	13.0	13.6
Some college	35.6	31.6	43.7	40.9
Bachelor's degree or more	45.9	56.4	41.7	44.2
Race/ethnicity (%)				
White	87.9	89.3	84.5	86.4
Black	3.7	3.7	8.4	7.1
Hispanic	3.1	3.2	3.7	3.3
Other or mixed race	5.2	3.8	3.4	3.2
Marital status (%)				
Married	92.3	92.8	74.0	75.4
Never married	2.0	1.3	7.1	7.3
Widowed/separated/divorced	5.7	5.9	18.9	17.4
Living with child's other parent (%)	89.9	89.8	64.6	65.9

### Comparison of Mothers and Fathers

In terms of their *communication about sexual topics *(Table 3), mothers generally talked with both sons and daughters more than fathers did. For daughters, the difference was especially pronounced and was statistically significant for a wide range of topics. For example, 59% of mothers said that they talked to their daughters about the biology of sex and pregnancy "a great deal" or "a moderate amount," compared with 43% of fathers. For sons, the difference between mothers' and fathers' communication was less pronounced: It was not statistically significant for any topic, but for three topics (the biology of sex and pregnancy, issues about dating and relationships, and the dangers of getting an STD) it did approach statistical significance (*q *< .10). Mothers were also more likely than fathers to report that they had asked or recommended to their children that they wait to have sex. This was true for both sons (69% of mothers vs. 60% of fathers) and daughters (77% of mothers vs. 68% of fathers).

**Table 3:** Parent-Child Communication about Sexual Topics, by Gender of Parent and Gender of Child

	Percent (%)				Overall *q*-values			
		
	Fathers		Mothers		By gender of parent		By gender of teen	
		
Communication	Sons	Daughters	Sons	Daughters	Mothers of sons vs. fathers of sons	Mothers of daughters vs. fathers of daughters	Fathers of sons vs. fathers of daughters	Mothers of sons vs. mothers of daughters
**How much talked about child's being sexually active and various topics (% a great deal or a moderate amount)**								
The biology of sex and pregnancy	43.7	42.8	52.1	59.0	0.069	0.000*	0.357	0.027*
Issues about dating and relationships	47.1	57.8	54.6	70.0	0.059	0.001*	0.021*	0.000*
Whether to wait to be sexually active until married	43.1	48.0	48.2	61.1	0.357	0.002*	0.442	0.001*
The moral issues of not having sex	43.8	48.3	48.6	58.5	0.457	0.029*	0.547	0.016*
The dangers of getting an STD	43.7	45.8	51.8	57.0	0.075	0.012*	0.849	0.176
The negative things that would happen if pregnant	48.0	52.4	49.8	58.1	0.860	0.350	0.517	0.055
The negative impact on his/her social life because of losing the respect of others	48.0	52.4	49.8	58.1	0.860	0.350	0.517	0.055
**Asked that child wait to have sex (% yes)**	60.4	67.8	69.3	76.8	0.012*	0.012*	0.069	0.020*

Note. STD = sexually transmitted disease.

* *q *< .05.

Mothers and fathers differed significantly on many factors associated with parent-child communication about sex (Table 4). In terms of their perception of the threat, mothers had more negative *attitudes toward teen sex*, in that they were more likely than fathers to believe that teenage sexual activity has harmful effects for both sons and daughters (e.g., with daughters, 50% of mothers strongly agreed vs. 38% of fathers). However, mothers and fathers did not differ significantly in their disapproval of their children being sexually active as teenagers. Mothers and fathers also did not differ in their *perception of the risk that their children would have sex as young teens *if asked by someone they were dating.

**Table 4:** Determinants of Parent-Child Communication about Sex, by Gender of Parent and Gender of Child

Determinants^a^	Percent (%)				Overall *q*-values			
		
	Fathers		Mothers		By gender of parent		By gender of teen	
		
	Sons	Daughters	Sons	Daughters	Mothers of sons vs. fathers of sons	Mothers of daughters vs. fathers of daughters	Fathers of sons vs. fathers of daughters	Mothers of sons vs. mothers of daughters
**Perception of threat**								

**Parent attitudes toward teen sex (% strongly agree)**								
Sexual activity is likely to have harmful effects for teens	32.5	38.4	37.6	50.0	0.012*	0.019*	0.001*	0.002*
Disapprove of child being sexually active as teenager	55.7	74.7	62.0	76.4	0.182	0.448	0.000*	0.000*
**Perceived risk child will have sex as young teen (% very likely or somewhat likely)**	35.0	12.4	28.3	12.6	0.125	0.796	0.000*	0.000*

**Expected outcomes of talking**								

**(% strongly agree)**								
Child will be less likely to have sex as a young teen	39.8	49.9	47.2	59.0	0.032*	0.122	0.046*	0.003*
Child would understand the benefits of waiting	27.8	37.4	33.0	49.2	0.193	0.020*	0.022*	0.000*
Child will listen^b^	23.4	25.1	27.7	34.9	0.601	0.002*	0.457	0.107
Child will not think you are judgmental	18.1	18.7	19.8	24.2	0.502	0.025*	0.168	0.228
Child will not think you are a hypocrite^b^	27.4	26.1	33.6	36.0	0.122	0.047*	0.171	0.860
Child would not rebel and want to have sex even more^b^	32.2	39.9	37.1	43.9	0.484	0.701	0.193	0.195

**Barriers and facilitators**								

**Shared activities with child (% at least once a week)**								
Gone shopping	43.7	33.6	56.7	62.8	0.000*	0.000*	0.015*	0.223
Do homework or school projects	59.8	59.9	78.5	75.2	0.000*	0.000*	0.860	0.517
Play a game or sport together	48.2	39.6	32.3	32.3	0.000*	0.185	0.071	0.417
Gone to a movie, sporting event, etc.	25.8	18.2	26.4	23.4	0.187	0.195	0.084	0.493
Watched an entire TV show together	74.7	70.1	75.9	77.3	0.796	0.163	0.616	0.918
Attend a party or family gathering together	21.8	21.7	20.5	23.9	0.493	0.054	0.839	0.502
**Closeness to child (% always)**								
You and child make decisions about child's life together	30.2	24.1	38.6	42.3	0.003*	0.000*	0.253	0.295
Child does not interfere with your activities^b^	35.6	32.1	43.2	43.7	0.214	0.007*	0.693	0.370
You get along well with child	51.7	46.9	46.4	40.4	0.353	0.276	0.676	0.168
You understand child^b^	17.2	12.3	13.8	15.1	0.539	0.693	0.457	0.488
You feel you can really trust child	42.5	46.5	42.3	47.3	0.940	0.841	0.792	0.539
**Conflict with child**								
In past 12 months, how often argued or had a fight (% 0-2 times)	46.5	41.6	30.7	29.9	0.000*	0.002*	0.659	0.697
**Self-efficacy: how sure can explain various topics (% completely sure)**								
Ways to have fun with a partner without having sex	39.0	36.6	48.2	47.5	0.031*	0.003*	0.115	0.616
Why he/she should wait until older to be sexually active	41.2	45.4	50.6	58.1	0.065	0.032*	0.350	0.163
How to say no	29.1	49.3	37.9	56.3	0.163	0.090	0.000*	0.000*
How to make a partner wait until child is ready	25.2	30.7	30.1	36.2	0.089	0.069	0.195	0.069
**Barriers to communication (% strongly disagree)**								
You don't know enough	62.2	62.9	76.0	77.0	0.000*	0.001*	0.796	0.604
It is difficult to find time to talk^b^	26.2	26.2	37.1	47.6	0.006*	0.000*	0.701	0.007*
It would be difficult for you to explain things	30.8	28.0	43.1	51.8	0.002*	0.000*	0.084	0.028*
**Beliefs about timing of talking (% strongly agree)**								
You are sure now is the right developmental time to talk	41.2	41.9	49.0	58.0	0.097	0.000*	0.909	0.072

^a ^In the table, the phrasing of some of the determinants is an abbreviated version of the actual questions in the questionnaire. The full wording is available from the authors upon request.

^b ^Reverse-coded.

* *q *< .05.

Mothers generally had more positive *expectations of the outcomes *of talking to their children about sexual activity than fathers did. With daughters, more mothers than fathers thought that their daughters would understand the benefits of waiting, would listen, and would not think the parent was being judgmental or hypocritical.With sons, the only difference was that more mothers than fathers thought that talking would reduce the likelihood that their sons would become sexually active as young teens (e.g., 47% of mothers vs. 40% of fathers strongly agreed).

Mothers generally had fewer barriers and more facilitators to communication than fathers did. Mothers *shared activities *with their children somewhat more than fathers did, especially with their daughters. Although mothers and fathers did not differ on most measures of *closeness*, one exception is that mothers were more involved with both sons and daughters in making decisions together about their lives. On the other hand, fathers reported less *conflict *with their children (both sons and daughters) than mothers (e.g., 69% of mothers reported having three or more arguments or fights in the past year with their sons, compared with 54% of fathers).

Mothers had higher *self-efficacy *than fathers for talking about some sexual topics with both sons and daughters. They also reported fewer *barriers to talking *with their children about sexual activity: with both sons and daughters, mothers were less likely than fathers to say they did not know enough, that it would be difficult to explain things, or that it was hard to find the time to talk. For example, with daughters, 52% of mothers strongly disagreed that it would be difficult to explain things, compared with 28% of fathers. Mothers were also more likely to say that they were sure it was the *right developmental time to talk *about sexual activity (e.g., with daughters, 58% of mothers vs. 42% of fathers strongly agreed).

### Comparison of Sons and Daughters

The gender of the child also made a difference in parent-child communications about sex. In terms of their *communication about sexual topics*, mothers talked more with daughters than with sons about numerous topics (see Table 3). Fathers talked with daughters more than with sons about just one topic: dating and relationships (58% of fathers of daughters had talked about it a great deal or a moderate amount with their child, compared with 47% of fathers of sons). Neither mothers nor fathers discussed any topics more with sons than with daughters. Mothers of daughters also asked or recommended that their child wait to have sex more often than mothers of sons (77% vs. 69%); among fathers, this difference approached significance (68% vs. 60%, *q *= .069).

Many factors influencing communication also differed between sons and daughters. In terms of *attitudes toward teen sex *(Table 4), both mothers and fathers were more likely to believe that sexual activity as a teenager would have harmful effects for daughters, as compared with sons, and parents were also more likely to disapprove of their daughters being sexually active as a teenager (e.g., among fathers, 75% strongly agreed that they disapproved for daughters, compared with 56% for sons). Both mothers and fathers perceived a higher *likelihood that sons would have sex as a young teen*, as compared with daughters (e.g., among mothers, 28% thought it was likely or very likely that sons would have sex if asked by someone they were dating, compared with 13% for daughters).

Both mothers and fathers also thought that some *expected outcomes of talking *about sexual topics would be more positive with daughters, as compared with sons. Specifically, compared with parents of sons, more parents of daughters thought that talking about delaying sexual activity would reduce the likelihood that their child would be sexually active and that their child would understand the benefits of waiting. For example, 49% of mothers of daughters strongly agreed that their child would understand the benefits of waiting, compared with 33% of mothers of sons. Other outcome expectations did not differ between sons and daughters.

Mothers and fathers reported almost no differences between sons and daughters in levels of *shared activities, closeness*, or *conflict*.

Both mothers and fathers had higher *self-efficacy *for explaining to daughters how to say no to sex, compared with sons (e.g., among fathers, 49% were completely sure they could explain it to daughters, compared with 29% for sons). In contrast, parents' confidence in their ability to explain three other topics did not differ significantly between sons and daughters. For mothers, two *barriers to talking *about sexual activity and waiting to have sex were less common with daughters than with sons: difficulty finding the time to talk and difficulty explaining things. For example, 52% of mothers of daughters strongly disagreed that it would be difficult for them to explain things, compared with 28% of mothers of sons. For fathers, there were no significant differences between sons and daughters.

## Discussion

Parent-child communication about sex differs markedly by the sex of the parent and the sex of the child. Similar to other studies [7,8,26,27], we found that mothers are generally more likely to talk to both their sons and their daughters about sex than fathers, and that mothers talk much more to their daughters than their sons. More surprisingly, we found that fathers were not more likely to talk to sons than daughters about any topic and that fathers were *more *likely to talk to daughters than sons about one topic--dating and relationships.

Research indicates that fathers' communication with their children about sex is important and that fathers accept that it is an important part of their parenting responsibilities [14,16]. However, the relatively low levels of communication by fathers found in this study suggest that fathers need additional support to talk to their children about sex. In addition, the low levels of communication by both mothers and fathers with their sons indicate that interventions designed to improve parent-child communication about sex should emphasize the importance of talking to sons about sex and help parents to overcome the barriers to communication with their sons.

Gender differences in communication can be better understood by looking at gender differences in the factors that may influence communication. To our knowledge, gender differences in these factors have not been investigated previously in a systematic manner. We found that mothers differ from fathers on a wide range of factors that would tend to make it easier for them to talk with their children about sex, and these differences tended to be more pronounced with daughters than with sons. Fathers' lower self-efficacy for talking about sex suggests that fathers need guidance about how to overcome the barriers to effective communication. Fathers may also need guidance about the right developmental time to talk to their children and reassurance that their sons and daughters will listen to them and value what they have to say--especially their daughters.

The differences in factors affecting parents' communication with their sons as compared to their daughters are striking in their reflection of societal double standards related to sexual activity. Parents of daughters (both mothers and fathers) are more disapproving of their children having sex than parents of sons, and they believe the consequences of sexual activity are more harmful for daughters. Moreover, parents of sons are less likely to believe that talking to their children about sex would be effective in encouraging them to delay sexual activity, and they are less confident that they can explain to their children how to say "no" to sex. To encourage parents to talk to sons as much as daughters about sex, it may be helpful to raise parents' awareness of the potential negative consequences for boys of early sexual activity and to counteract the feeling of the inevitability of boys having sex.

A strength of this study is that it uses data from a large, national sample. To maximize the representativeness of the sample, the Knowledge Networks panel uses random-digit-dialing methodologies for recruitment and includes both Internet and non-Internet households. Nonetheless, the sample includes a higher proportion of whites and has higher levels of education than the United States as a whole, so it does not fully represent the U.S. population. Parent-child communication about sex has been found in previous research to differ by race/ethnicity and by socioeconomic status [4], so results may differ for samples with a higher proportion of minorities or lower levels of education. Another limitation of the sample is that it included only parents who co-resided with their children. Therefore, findings cannot be generalized to parents who are not living with their children. Although we did not control for race/ethnicity or socioeconomic status in our analyses, there were no significant differences across the four gender groups in terms of their racial/ethnic composition or socioeconomic status. Therefore, the differences observed in factors affecting parent-child communication cannot be attributed to differences in race/ethnicity or socioeconomic status.

## Conclusions

In summary, this study confirms findings from previous studies showing differences in parent-child communication about sexual topics, by both gender of the parent and gender of the child, using data from a large national sample. More importantly, it identifies gender differences in factors that may influence parent-child communication about sex not previously investigated. Understanding these differences can help to inform the design of interventions targeting each gender group. Fathers in particular need support to overcome numerous barriers to communication with both sons and daughters, and mothers and fathers would benefit from interventions to increase their understanding of the feasibility and the importance of encouraging sons in particular to delay sexual activity.

## Competing interests

The authors declare that they have no competing interests.

## Authors' contributions

EW conceptualized the study, led the analysis, and wrote the text of the paper. HK advised on the conceptualization of the study, analysis of the data, and presentation of the results. HK also reviewed and edited the text. Both authors read and approved the final manuscript.
